# Analysis of Yeast Killer Toxin K1 Precursor Processing via Site-Directed Mutagenesis: Implications for Toxicity and Immunity

**DOI:** 10.1128/mSphere.00979-19

**Published:** 2020-02-12

**Authors:** Stefanie Gier, Manfred J. Schmitt, Frank Breinig

**Affiliations:** a Molecular and Cell Biology, Saarland University, Saarbrücken, Germany; b Center of Human and Molecular Biology (ZHMB), Saarland University, Saarbrücken, Germany; University of Georgia

**Keywords:** K1, killer toxin, yeast viral toxin, *Saccharomyces cerevisiae*

## Abstract

K1 represents a heterodimeric A/B toxin secreted by virus-infected Saccharomyces cerevisiae strains. In a two-staged receptor-mediated process, the ionophoric activity of K1 leads to an uncontrolled influx of protons, culminating in the breakdown of the cellular transmembrane potential of sensitive cells. K1 killer yeast necessitate not only an immunity mechanism saving the toxin-producing cell from its own toxin but, additionally, a molecular system inactivating the toxic α subunit within the secretory pathway. In this study, different derivatives of the K1 precursor were constructed to analyze the biological function of particular structural components and their influence on toxin activity as well as the formation of protective immunity. Our data implicate an inactivation of the α subunit during toxin maturation and provide the basis for an updated model of K1 maturation within the host cell’s secretory pathway.

**IMPORTANCE** The killer phenotype in the baker’s yeast Saccharomyces cerevisiae relies on two double-stranded RNA viruses that are persistently present in the cytoplasm. As they carry the same receptor populations as sensitive cells, killer yeast cells need—in contrast to various bacterial toxin producers—a specialized immunity mechanism. The ionophoric killer toxin K1 leads to the formation of cation-specific pores in the plasma membrane of sensitive yeast cells. Based on the data generated in this study, we were able to update the current model of toxin processing, validating the temporary inactivation of the toxic α subunit during maturation in the secretory pathway of the killer yeast.

## Abstract (precis)

The killer phenotype in the baker’s yeast Saccharomyces cerevisiae relies on two double-stranded RNA viruses that are persistently present in the cytoplasm. As they carry the same receptor populations as sensitive cells, killer yeast cells need—in contrast to various bacterial toxin producers—a specialized immunity mechanism. The ionophoric killer toxin K1 leads to the formation of cation-specific pores in the plasma membrane of sensitive yeast cells. Based on the data generated in this study, we were able to update the current model of toxin processing, validating the temporary inactivation of the toxic α subunit during maturation in the secretory pathway of the killer yeast.

## INTRODUCTION

The killer phenotype is a wide-spread phenomenon in various yeast strains and characterized by the secretion of proteinaceous compounds, so-called killer toxins, which are lethal to sensitive yeast strains. In the case of the baker’s yeast Saccharomyces cerevisiae, four virally encoded killer toxins (K1, K2, K28, and Klus) have been described based on their killing properties and the lack of cross-immunity (1). Killer yeast secreting these toxins are infected with two distinct double-stranded RNA viruses, M and L-A, persistently present in the cytoplasm of the host cell. Whereas the M virus encodes the respective toxin, L-A ensures the replication and maintenance of both mycoviruses by encoding the major capsid protein Gag and the RNA-dependent RNA polymerase Pol, which is expressed as a Gag-Pol fusion protein (2, 3). Primarily translated as precursor proteins (preprotoxin [pptox]), each killer toxin undergoes further maturation in the secretory pathway of its particular host cell. Mature K1 and K28 toxin molecules resemble classical A/B toxins consisting of one toxic α subunit and one β subunit, which is essential for target cell binding (3). Despite their different modes of action, the structural compositions as well as the processing of these killer toxin precursors are similar. Each preprotoxin consists of an N-terminal signal peptide (SP; preregion) critical for the import into the lumen of the endoplasmic reticulum (ER) followed by a proregion whose biological function has not yet been elucidated. Both major toxin subunits are separated by an additional γ component, which probably acts as an internal chaperone. After cleavage of the preregion and potential N-glycosylation of the γ subunit in the ER lumen, α and β are covalently linked via one single disulfide bond, and the toxin undergoes additional processing steps in the late Golgi compartment, involving the peptidases Kex2p and Kex1p (4). Eventually, the mature heterodimeric A/B toxin is packed into vesicles and secreted into the environment (5). Both killer toxins, K1 and K28, act in a receptor-mediated two-staged process, whereas the first step involves a rapid and energy-independent binding to the respective primary receptor in the cell wall (6, 7). In the case of K28, the heterodimeric toxin binds to its membrane receptor Erd2p, thereby parasitizing the retrograde transport machinery of the target cell. After translocation from the ER to the cytoplasm, the α subunit induces a cell cycle arrest in sensitive yeast (8, 9). In contrast, K1 toxin first binds to the β-1,6-glucan fraction within the yeast cell wall and is subsequently transferred to the plasma membrane level, where it interacts with its secondary receptor Kre1p (10, 11). Early studies on K1 were able to demonstrate the strong hydrophobic character of the α subunit and the ability of the toxin to form pores into artificial membranes leading to a breakdown of the transmembrane gradient and death of sensitive cells (12, 13). The current model of toxin action allows both a direct insertion into the membrane as well as further interaction with one or more so far unknown primary effector protein(s) (10). Remarkably, killer toxin-producing yeast cells need a particular mechanism granting immunity against their own toxin. On a molecular level, this mechanism has only been elucidated for K28 and is essentially linked to the posttranslational import of the toxin precursor, which interacts with reinternalized mature toxin molecules in the cytosol, blocking their action (14). Based on the hydrophobicity of the K1 preregion, cotranslational ER import of the precursor molecule is proposed, which would exclude a comparable immunity mechanism; nevertheless, the preprotoxin and its import into the secretory pathway are critically involved in the formation of intrinsic immunity (15, 16). A reduction of the plasma membrane Kre1p population in K1 killer cells was suggested to confer immunity due to complexation of Kre1p and the K1 protoxin in the secretory pathway, leading to vacuolar degradation of this complex (17, 18). However, we were recently able to show that the plasma membrane receptor Kre1p is not involved in the immunity mechanism by intracellularly expressing different K1 derivatives in a Δ*kre1* null mutation background (15). Although a previously conducted comprehensive mutational analysis of the toxin precursor was able to show the importance of the γ subunit in K1 immunity, neither the exact mechanism of the toxic effect nor the intrinsic immunity have been completely elucidated to date (19). Based on existing hypotheses of K1 maturation, we constructed various K1 derivatives mutated in different toxin subunits to analyze the impact of these structural components in K1 biology. Via intracellular expression of these toxin constructs, we were able to further describe the biological function of specific steps in the K1 maturation process. Moreover, based on the generated data, we postulate a model suggesting the inactivation of the toxic α subunit in dependency of the proregion and γ region, thereby refining the current model of K1 precursor processing.

## RESULTS

### Mutation of the K1 preproregion.

**FIG 1:**
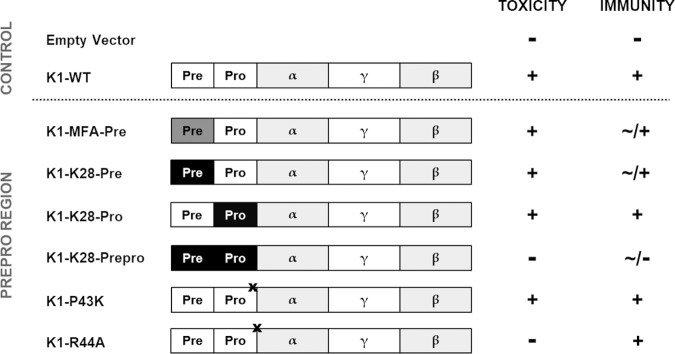
Effects of mutations within the K1 preproregion on toxicity and the formation of immunity. Secretion of biological active K1 derivatives, as well as the mediation of immunity, was analyzed via MBA diffusion assay (see Fig. S1 in the supplemental material). Wild-type toxicity, as well as functional immunity, is marked +, and loss of toxicity/immunity is marked −. Incomplete immunity (∼) is defined as the formation of a zone with reduced cell growth. K28-specific subunits are illustrated in black; MFA preregion is marked in gray; the position of point mutations is marked by a black X. Representative images of the respective killing zones are depicted in Fig. S2A.

The N-terminal preregion present in K1 and K28 precursors is necessary for ER import and, therefore, critical for further maturation of the particular toxin. We have previously shown that the posttranslational import of K28 pptox into the secretory pathway has direct implications for the immunity mechanism, saving the toxin-producing cell from its own reinternalized toxin (14). To extend the current understanding of the precursor’s ER import type, we systematically mutated the K1 preregion (amino acids [aa] 1 to 26) with the corresponding subunits of K28 (K1-K28-Pre) and the “mating type factor **a**” (MFA; K1-MFA-Pre), respectively, which are both posttranslationally imported proteins (Fig. 1). In addition to the ER import sequence, we analyzed the consequences of different mutations of the K1 proregion (aa 27 to 44). This short but hydrophobic sequence, whose exact molecular function has not yet been elucidated, is removed in a Kex2p-dependent manner in late Golgi compartments of the killer yeast. However, the wild-type enzyme cleavage site (PR_44_-E_45_A) represents a moderate to weak recognition signal for the endopeptidase Kex2p, preferably acting at dibasic amino acid sequences. Thus, besides the complete substitution of the proregion with the corresponding K28 sequence alone (K1-K28-Pro) or in combination with the preregion [K1-K28(SS)], point mutations affecting the efficacy of the Kex2p cleavage site at the N terminus of the toxic α subunit were introduced, creating either a strongly impaired (K1-R_44_A) or improved (K1-P_43_K) enzyme recognition site (Fig. 1). All derivatives were cloned into the multicopy vector pYES2.1 and transformed in the sensitive yeast strain BY4742. Subsequently, K1 toxicity, as well as the mediation of functional immunity, was analyzed via methylene blue agar (MBA) diffusion assay (see Fig. S1 and S2 in the supplemental material). Although the exchange of the K1 preregion with the respective sequence of K28 (K1-K28-Pre) or MFA (K1-MFA-Pre) slightly diminished immunity against extracellularly applied K1 toxin, no reduction in biological activity (i.e., secretion of biologically active toxin) was detected. Likewise, a wild-type phenotype regarding toxin secretion and immunity was observed after substitution of the K1 proregion by the corresponding K28 sequence (K1-K28-Pro). The combined replacement of the wild-type pre- and proregions by the K28-specific subunits, however, resulted in a loss of toxicity [K1-K28(SS)]. Concerning the point mutations altering the recognition site of Kex2p of the preregion, transformants expressing a K1 derivative with an enhanced enzyme recognition site showed a wild-type phenotype (K1-P_43_K). In clear contrast, the impairment of this cleavage site by substitution of arginine with the unipolar amino acid alanine resulted in a complete loss of toxicity, whereas respective transformants remained entirely immune against externally applied K1 toxin (K1-R_44_A) (Fig. 1).

10.1128/mSphere.00979-19.1 FIG S1 Phenotypes induced by intracellular expression of different K1 derivatives. (Left) Toxicity. Secretion and biological activity of the K1 derivatives were qualitatively analyzed via MBA diffusion assay against S. cerevisiae BY4742. Therefore, 10^6^ intact cells of the sensitive strain were embedded into MBA medium (sc galactose, pH 4.7). Subsequently, 10^6^ cells of the transformants were spotted onto the agar plates. Biological activity was determined by the formation of killing zones (+); loss of toxin secretion/activity is indicated as −. (Right) Immunity. Functional immunity was analyzed by embedding 10^6^ cells of each transformant into ura d/o galactose-containing MBA (pH 4.7) and applying 1,000 AU K1 toxin concentrate. Formation of wild-type functional immunity is labeled +, loss of immunity as −. A zone of reduced growth with no clear killing zone was defined as incomplete functional immunity and is as ∼. Download FIG S1, TIF file, 0.2 MB. Copyright © 2020 Gier et al. 2020 Gier et al. This content is distributed under the terms of the Creative Commons Attribution 4.0 International license.

10.1128/mSphere.00979-19.2 FIG S2 Killing zones generated by K1 derivatives with mutated preproregions (A) or mutations in the γ subunit (B); 10^6^ cells of the sensitive strain BY4742 were embedded into galactose-containing MBA (pH 4.7), and 10^6^ cells of the indicated transformants were spotted onto the agar. The formation of killing zones was documented after 3 days (20°C), and representative triplicates are depicted. Download FIG S2, TIF file, 0.5 MB. Copyright © 2020 Gier et al. 2020 Gier et al. This content is distributed under the terms of the Creative Commons Attribution 4.0 International license.

**FIG 2:**
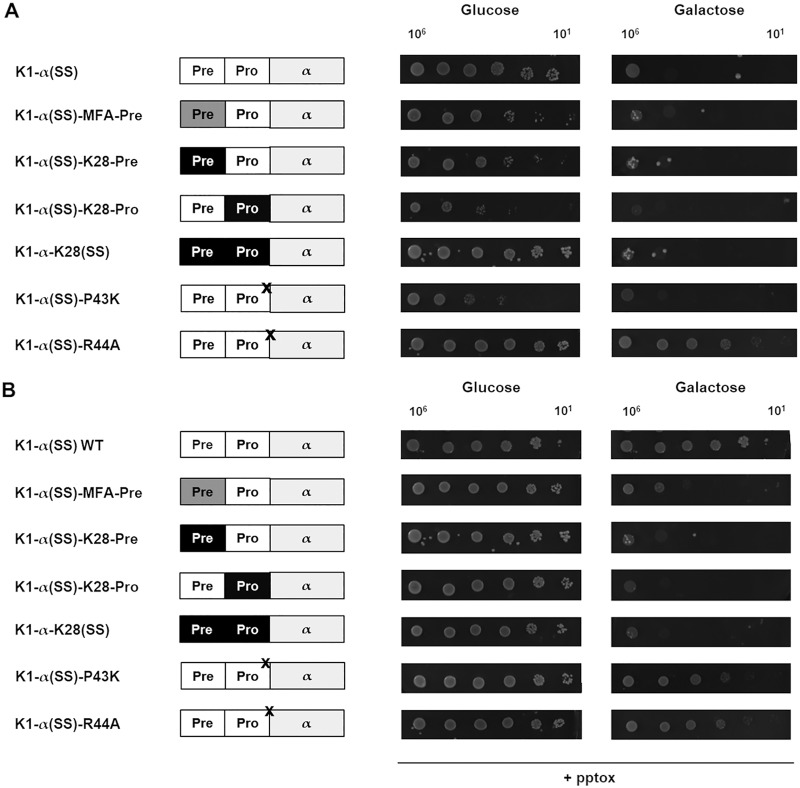
(A) Intracellular expression of lethal constructs with mutated preproregions. Cells of the sensitive S. cerevisiae strain BY4742 were transformed with the indicated K1 α derivatives, and logarithmic dilutions of 10^6^ to 10^1^ cells were spotted onto appropriate agar plates containing either glucose (noninducing conditions, left) or galactose (inducing conditions, right) as carbon source. Growth was documented after 3 days (30°C), and representative results are depicted (*n* = 15). (B) Cotransformation of mutated α derivatives with wild-type toxin precursor. BY4742 cells were cotransformed with the indicated α derivatives and the wild-type K1 precursors molecule (pYX242). Respective transformants were spotted in logarithmic dilutions (10^6^ to 10^1^ cells) onto ura/leu d/o agar plates (glucose: noninducing conditions, left; galactose: inducing conditions, right). Cell growth was documented after 3 days (30°C), and representative results were visualized (*n* =15). K28-specific subunits are illustrated in black, MFA preregion is depicted in gray, and point mutations are marked by a black X.

Furthermore, we analyzed potential effects of the described mutations in the preproregion on the pore-forming abilities of the toxin. Corresponding to previously described lethal constructs consisting only of the toxic α subunit, truncated K1 derivatives containing the different preproregions were obtained via PCR and expressed in the sensitive strain BY4742 (15). The potential impact of the mutations on the growth of the transformants (i.e., the suicidal phenotype) was analyzed via serial dilution assay (Fig. 2A). The partial or full substitution of the K1 preproregion with either the corresponding sequence of killer toxin K28 [K1-α(SS)-K28-Pre, K1-α(SS)-K28-Pro, and K1-α-K28(SS)] or MFA [K1-α(SS)-MFA-Pre] thereby induced a wild-type growth defect in the respective transformants, indicating no effect of the mutations on the pore-forming ability of α. Interestingly, even under noninducing conditions (glucose), substantial growth defects were observed for these derivatives. Likewise, the enhancement of the Kex2p cleavage of the proregion [K1-α(SS)-P_43_K] had no effect on the suicidal phenotype, whereas the transformants expressing lethal α constructs with an impaired enzyme recognition site [K1-α(SS)-R_44_A] were able to grow under inducing conditions (Fig. 2A). Potential interference of these mutated α derivatives in the intrinsic immunity mechanism was further analyzed via serial dilution assay after cotransformation with the wild-type K1 preprotoxin (Fig. 2B). Regarding the α constructs with fully or partially substituted preproregions [K1-α(SS)-K28-Pre, K1-α(SS)-K28-Pro, K1-α-K28(SS), and K1-α(SS)-MFA-Pre], a distinct loss in immunity was observed, which is generally granted by the precursor molecule in the secretory pathway, with no considerable difference between the various lethal constructs. In contrast, no modification of the Kex2p recognition site [K1-α(SS)-P_43_K or K1-α(SS)-R_44_A] influenced the formation of functional immunity under inducing conditions, creating a wild-type phenotype (Fig. 2B).

### Mutation of the K1 γ subunit.

**FIG 3:**
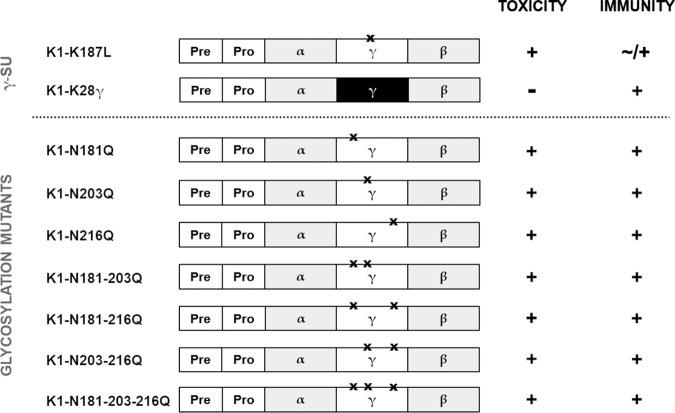
Effects of mutations within the K1 γ subunit. Depicted is the summary of the observed phenotypes regarding toxicity and immunity against extracellularly applied K1 toxin after expressing the indicated K1 derivatives in the sensitive strain BY4742. A wild-type phenotype (biological activity and functional immunity) is indicated as +, whereas no toxicity and loss of immunity are labeled −. Incomplete mediation of immunity (∼) is defined by a reduction in growth but no distinct killing zone. Point mutations are indicated by a black X, and substitution with K28-specific sequences are illustrated in black. Images of representative results are depicted in Fig. S2B.

We also analyzed the potential effects of individual point mutations in the K1 γ subunit by either destroying the glycosylation sites (N_181_, N_203_, and N_216_) or the intramolecular Kex2p recognition sequence (KR_188_). Additionally, a complete substitution of γ with the corresponding K28 subunit was conducted (K1-K28γ). After the expression of the latter, no biological activity against intact sensitive cells was observed, but the respective transformants were still immune against externally applied K1 toxin. In contrast, cells expressing a K1 derivative with a nonfunctional intramolecular Kex2p cleavage site in γ (K1-K_187_L) secreted biologically active toxin but were not entirely immune when additional wild-type toxin was applied externally. Remarkably, mutation of the glycosylation sites had no effect on the biological activity of the respective secreted K1 derivatives nor on the ability to mediate functional immunity, and all constructs showed a wild-type phenotype (Fig. 3 and S2).

**FIG 4:**
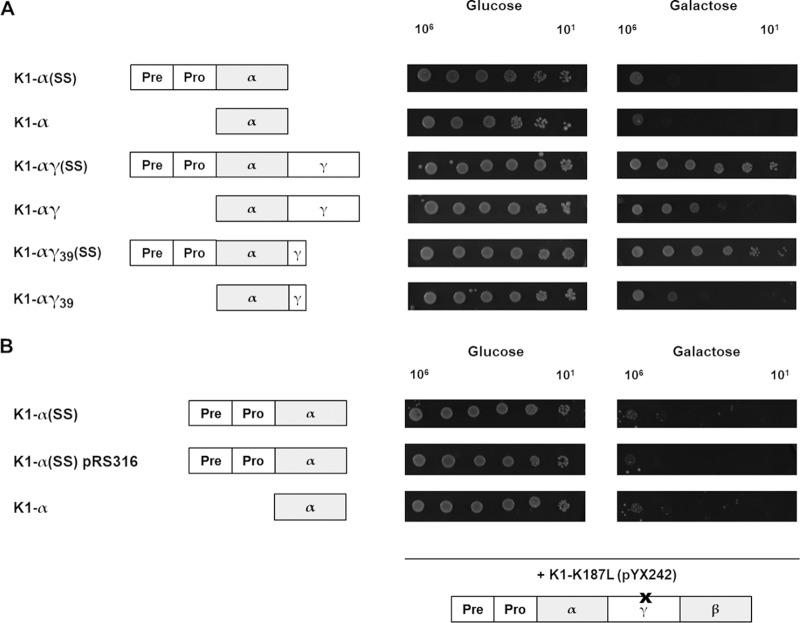
(A) Intracellular expression of αγ derivatives. After transformation of S. cerevisiae with the indicated K1 αγ derivatives (with or without native K1 preproregion [SS]), effects on cell growth were analyzed via serial dilution assay; 10^6^ to 10^1^ cells of the respective transformants were spotted onto glucose-containing (noninducing, left) or galactose-containing (inducing, right) agar plates. After 3 days at 30°C, growth was documented; depicted are representative results (*n* = 30). (B) Cotransformation of point-mutated toxin precursor with wild-type α constructs. Cotransformation of BY4742 cells was conducted using K1 α constructs with (α[SS]) or without the preproregion (α) and a point mutated K1 pptox derivative with a nonfunctional Kex2p cleavage site (K1-K_187_L in pYX242). Lethal constructs were expressed either in the multicopy vector pYES2.1 or a low-copy-number vector pRS316. Transformants were spotted onto ura/leu d/o agar plates (glucose: noninducing, left; galactose: inducing, right). After incubation (3 days, 30°C), cell growth was documented, and representative results were visualized (*n* = 5).

As previous studies already implied the importance of the γ subunit in the formation of immunity (19), we constructed α derivatives that were either C-terminally extended by the complete γ sequence [K1-αγ(SS) and K1-αγ] or the N-terminal 39 amino acids of this subunit [K1-αγ_39_(SS) and K1-αγ_39_], which would remain after intramolecular cleavage by Kex2p. Intracellular expression of these constructs led to a diminution of the suicidal phenotype, showing the most significant impact of when the respective constructs were imported into the secretory pathway (Fig. 4A). This phenotype was also mirrored in cotransformational experiments with the wild-type toxin precursor and a truncated version (K1-tox lacking the preproregion) showing the potential of αγ(SS) derivatives to grant immunity completely (see Fig. S3). The importance of the intramolecular Kex2p recognition site was further analyzed by subcloning of the point-mutated full-length toxin precursor (K1-K_187_L) into the vector pYX242. After coexpression with different wild-type α constructs [α and α(SS)] in the multicopy vector pYES2.1 or a low-copy-number vector pRS316, respectively, cellular growth of the respective transformants was analyzed via serial dilution assay (Fig. 4B). Interestingly, and in sharp contrast to the wild-type precursor, no immunity was established by the mutated K1 precursor derivative independent of the cotransformed lethal construct.

10.1128/mSphere.00979-19.3 FIG S3 Coexpression of αγ constructs with the wild-type toxin precursor. Cotransformation of BY4742 cells was conducted using K1 αγ constructs with (α[SS]) or without the preproregion (α) and the wild-type K1 pptox derivative (pptox; left). A truncated version (K1-tox; right) lacking the preproregion was used as a control. Transformants were spotted onto galactose-containing ura/leu d/o agar plates (glucose: noninduced control, not shown). Cell growth was documented after 3 days (30°C), and representative results were visualized (*n* = 30). Download FIG S3, TIF file, 0.1 MB. Copyright © 2020 Gier et al. 2020 Gier et al. This content is distributed under the terms of the Creative Commons Attribution 4.0 International license.

## DISCUSSION

**FIG 5:**
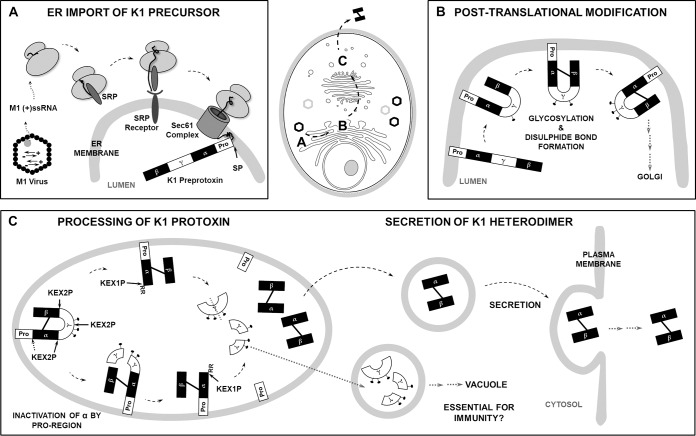
Schematic illustrations of K1 killer toxin processing in the secretory pathway, and the formation of immunity of a killer yeast cell. (A) ER import of toxin precursor molecule. (+)ssRNA molecules are translated by host cell ribosomes, and resulting precursors are cotranslationally imported into the ER, where the preregion is cleaved by the signal peptidase complex (SP). (B) Posttranslational modifications of the K1 protoxin. In the host cell ER, the major toxin subunits α and β are covalently linked via one disulfide bond, and the γ subunit is glycosylated. Subsequently, the protoxin is anterogradely transported to the Golgi apparatus. (C) Processing of the K1 protoxin to the mature heterodimer. In a late Golgi compartment, the protoxin is further processed by the endopeptidase Kex2p, which successively generates the N terminus of β as well as the N and C termini of α. During this process, the toxic α subunit is potentially kept inactivated by the membrane-bound proregion and parts of the γ subunit. Subsequently, the newly exposed dibasic arginines at the α C terminus are proteolytically removed by the carboxypeptidase Kex1p. The mature K1 heterodimer is transported to the plasma membrane and secreted into the extracellular medium. Previous studies additionally imply a transport of the γ sequence to the vacuole, which is important for the mediation of immunity. (Data from references 24 and 35 were used to create this figure.)

K1 is an ionophoric A/B toxin produced and secreted by virus-infected Saccharomyces cerevisiae strains killing sensitive yeast cells by destroying cytoplasmic membrane structures and functions. Similarly to mammalian precursor proteins such as insulin, K1 is initially translated as a preprotoxin with a distinct domain organization, undergoing various posttranslational modifications critical for its biological activity. Although many studies highlighted the importance of the toxin precursor’s import into the secretory pathway for the mediation of functional immunity in the toxin-producing cell, it is still unclear if the K1 preprotoxin is post- or cotranslationally transported into the ER (20, 21). Based on their fast growth, yeast and bacterial cells mostly prefer posttranslational import of peptides and proteins into the ER, as the number of *Sec* complexes/equivalents in the cell is limited. Cotranslational import only occurs for highly hydrophobic proteins that would otherwise accumulate in the cytoplasm (22, 23). Previous sequence analyses revealed several highly hydrophobic regions within the ionophoric toxin, predestining K1 for cotranslational ER import (24). Besides, the characterization of functional immunity against the killer toxin K28 illustrated that the underlying mechanism is tightly connected to the posttranslational ER import of the toxin precursor molecule (14). In an approach to analyze the ER transport of K1 preprotoxin *in vitro*, we substituted the preregion of the toxin with the corresponding sequence of K28 or MFA to ensure posttranslational import (14, 25). Although the respective transformants were able to secrete biologically active toxin, a reduction in cellular growth was observed after extracellular application of wild-type K1 toxin, indicating an impairment in the mediation of intrinsic immunity. Additional cotranslational experiments conducted using chimeric lethal constructs (derivatives lacking the γ and β subunit) and the wild-type K1 preprotoxin resulted in complete growth inhibition of the transformants under inducing conditions. This observation further strengthened the hypothesis of cotranslational import of the K1 killer toxin while concurrently excluding an immunity mechanism similar to that for K28 (Fig. 5A).

After cleavage of the preregion by the signal peptidase complex in the ER, the newly generated N terminus consists of the so-called proregion, which is sometimes referred to as the δ subunit. Based on its highly hydrophobic properties, this short peptide is also proposed to be anchored within the luminal membrane during the maturation process until it is removed in a Kex2p-dependent manner in late Golgi compartments, generating the mature N terminus of α (20, 24, 26). However, the endopeptidase recognition site comprises only one charged amino acid residue (Arg) instead of the favorable dibasic residues, lowering the cleavage efficiency (27). In an attempt to analyze the importance of propeptide cleavage as well as its general function in the K1 maturation process, we altered this Kex2p cleavage site, either creating a highly efficient dibasic (ProArg→LysArg) or an ineffective (ProArg→ProAla) recognition site. Interestingly, the latter completely diminished the toxic effect of α, which was also reproduced using lethal constructs. In addition to and in accordance with a previous study, we substituted the complete proregion with the corresponding sequence of K28 (14). The expression of these K1 derivatives (full-length toxin or α derivatives) had no influence on the secretion of biologically active toxin, functional immunity, or the development of the characteristic suicidal phenotype. However, as soon as both the pre- and the proregion of the killer toxin were substituted with the respective K28 subunits, a loss of toxicity and a diminution of immunity after extracellular application of wild-type K1 toxin were detected. In contrast to K1, the K28 proregion is even shorter and does not exhibit the same hydrophobicity profile but a comparable weak C-terminal Kex2p cleavage site. Based on our data, the K28 proregion does not seem to affect the general maturation process of K1 or the toxicity of the α subunit. However, the combined substitution of the whole preproregion led to an insufficient formation of immunity, implicating an inadequate number of precursor molecules in the secretory pathway to counteract the toxic effect, probably due to the posttranslation import of the mutated K1 preprotoxin. Taken together, we propose an involvement of the K1 proregion not only in the correct folding of the toxin heterodimer during its processing within the host cell but also in the inactivation of the toxic α subunit to the point of secretion.

In the first maturation steps occurring in the ER of the host cell, α and β are linked via one disulfide bond, and glycosylation of the γ sequence is proposed (4) (Fig. 5B). In this study, we analyzed the impact of successive mutations of these N-glycosylation recognition sites (Asn→Gln) on K1 toxicity and the intrinsic immunity mechanism. In contrast to an earlier study reporting a complete stop in toxin secretion after the application of tunicamycin (28), the substitution of the glycosylation sites had no impact on the toxin’s activity. By blocking the first step of N-glycosylation, tunicamycin induces ER stress via the accumulation of misfolded proteins, initiating the cellular unfolded protein response (29). Thus, the observed decrease in K1 secretion could be a consequence of a more general reduction in protein translation to restore protein homeostasis (30). Additionally, the peptidases Kex2p and Kex1p crucial for toxin maturation are heavily glycosylated proteins, and significant misfolding of these enzymes would lead to a similar phenotype that can be observed for Δ*kex2* deletion mutants (31, 32). Although glycosylation of the K1 protoxin has been verified via Western blotting and endoglycosidase H treatment, we propose that this posttranslational modification has no impact on the general K1 toxicity and might be just facilitating its processing (28) (Fig. 5B).

In addition to the chaperone-like function, the γ subunit has also been shown to be crucial for the formation of immunity (19). We analyzed a possible effect of this sequence on the secretion of biologically active toxin as well as the establishment of functional immunity after substitution with the corresponding subunit of K28. Although the sequences of both killer toxins show no sequence similarities, complete mediation of intrinsic immunity was observed for the respective transformants. Therefore, it could be hypothesized that only the physicochemical properties of the C-terminal elongation of α are important for the immunity mechanism independent from the exact amino acid sequence. However, yeast expressing this construct were not able to secrete a biologically active toxin, which was also reported for the analogous exchange in the K28 killer toxin in a previous study and is likely based on the distinct structural alteration of the killer toxins (14). To further examine the role of γ in K1 biology, we impaired the intramolecular Kex2p cleavage site. Although no reduction in toxicity was detected, a minor impairment in the formation of immunity was observed when K1 toxin was added externally. This disruption in the immunity mechanism was even more evident after coexpression of the mutated precursor toxin with different lethal constructs, indicating a significant role of Kex2p-dependent processing of the γ subunit as soon as free α molecules are present in the Golgi. Therefore, we analyzed αγ derivatives consisting of either the complete γ sequence or a truncated version of 39 amino acids resembling the subunit after intramolecular Kex2p cleavage. After intracellular expression of these constructs, the complete mediation of immunity was solely granted for derivatives bearing an ER import signal, thus being able to enter the secretory pathway. The results of this study not only support previous findings showing the ability of the first 31 amino acids of γ subunit’s N terminus to confer full immunity but also highlight the importance of Kex2p-dependent processing and cleavage of γ (19).

In summary, we were able to strengthen and expand the current model of K1 toxin processing within the secretory pathway of the host cell. Based on the phenotypes of point-mutated K1 derivatives with altered Kex2p recognition sites in different toxin subunits, we postulate a model of K1 toxin maturation based on inactivation of the toxic α subunit by the proregion and the γ subunit. We suggest a successive proteolytical removal of the different protoxin regions in late Golgi compartments, thereby protecting the toxin-producing cell (Fig. 5C). The mature toxin heterodimer is further transported to the plasma membrane, where it is eventually secreted into the extracellular environment. Our data link different findings of previously conducted studies and provide valuable basics for future experiments. Moreover, we were able to describe a critical role of the N-terminal proregion in the maturation process for the first time. Future experiments could focus further on the ability of different N-terminal peptide sequences to inactivate the toxic K1-α subunit. For example, the chimeric K1 to K28 derivatives described in this study could be further analyzed by altering the Kex2p cleavage site of the K28 proregion. This could clarify if the observed phenotype is based on steric inhibition only or if a specific sequence-dependent mechanism is involved.

## MATERIALS AND METHODS

### Strains, plasmids, and DNA manipulation.

Escherichia coli strain TOP10F′ {F′ [*lacI*^q^ Tn*10* (Tet^r^)] *mcrA* Δ(*mrr*-*hsdRMS*-*mcrBC*) Φ80*lacZ*ΔM15 Δ*lacΧ74 recA1 araD139* Δ(*ara-leu*)7697 *galU galK rpsL* (Str^r^) *endA1 nupG*} (Invitrogen) was used for cloning and amplification of all constructs. Cells were grown at 37°C in LB medium (1% tryptone, 0.5% yeast extract, and 0.5% sodium chloride) supplemented with 100 μg/ml of ampicillin when necessary. All K1 derivatives were generated by PCR and cloned into pYES2.1/V5-HIS-TOPO vector (Thermo Scientific) as described before (15) using the listed primers (Table 1); subcloning was conducted into the yeast expression vectors pYX242 and pRS316. The K1-sensitive Saccharomyces cerevisiae strain BY4742 (*MAT*α *his3*Δ1 *leu2*Δ0 *lys2*Δ0 *ura3*Δ0) was obtained from DharmaconGE. K1 toxin was produced by S. cerevisiae strain T158c (*MAT*α *his4C-864*). Cells were grown at 30°C in standard growth medium (1% yeast extract, 2% peptone, 2% glucose). Toxin production and concentration were determined as described before (33).

**TABLE 1:** Primers used to generate K1 toxin derivatives

Primer	5′→3′ sequence^*a*^
5′ K1-pptox	ctc gag gaa ttc ATG ACG AAG CCA ACC CAA GTA TTA GTT AGA
5′ K28-pre	ctc gag gaa ttc ATG ATG GAG AGC GTT TCC TCA TTA TTT AAC A
5′ MFA-pre	ctc gag gaa ttc ATG AGA TTT CCT TCA ATT TTT ACT GCA G
5′ K1-α	ctc gag gaa ttc ATG GAA GCG CCG TGG TAT GAC AAG ATC TG
3′ K1-pptox	aag ctt cct agg gcggccgc CTA GTG GCC TGT GTC
3′ K1-α	aga tct gtc gac cct agg aag ctt TTA AGC AAC GGT AGC GCC ATT AGG ATC TGC
3′ K1-αR	aga tct gtc gac cct agg aag ctt TTA ACG AGC AAC GGT AGC GCC ATT AGG AT
3′ K1-αRR	aga tct gtc gac aag ctt TTA ACG ACG AGC AAC GGT AGC GCC ATT AGG AT
3′ K1-γ	aga tct gtc gac aag ctt TTA ACG CTT GGC CAC TGC TGG AAT G
3′ K1-γ(39)	aga tct gtc gac aag ctt TTA GAC ATA TTG TGA TGC GTT AGC TGG GAG TAT ACT AAT AC

a Restriction sites are illustrated as lowercase letters, and start and stop codons are underlined.

### Yeast transformation and selection.

For *in vivo* expression of K1 derivatives, Saccharomyces cerevisiae strain BY4742 was transformed by the standard lithium acetate method (34) and cultivated using appropriate selective media.

### Characterization of suicidal phenotype.

The lethal effect of intracellular expressed K1 derivatives was analyzed by performing serial dilution assays. Colonies of the respective transformants were inoculated in standard synthetic medium containing 2% glucose. After incubation for 16 h at 30°C, the cell number was determined, and 10^7^ cells were harvested. Cells were washed twice in synthetic medium containing 2% raffinose to eliminate remaining glucose. Subsequently, logarithmic dilutions were performed, and 10^1^ to 10^6^ cells were spotted onto uracil (ura) dropout (d/o) and ura/leucine (leu) d/o (cotransformation) agar plates, respectively, containing either glucose (control) or galactose (inducing conditions). Plates were incubated for 3 days at 30°C, and cellular growth was documented.

### Analysis of toxin activity and mediation of immunity.

Biological activity of secreted K1 toxin was checked via MBA diffusion assay. Therefore, 10^6^ cells of the sensitive strain BY4742 were embedded into MBA agar (synthetic complete [sc] galactose, pH 4.7), and 10^6^ cells of the respective transformant were spotted onto the plate. The mediation of functional immunity was analyzed by embedding 10^6^ cells of each transformant into MBA agar (ura d/o galactose, pH 4.7), and applying 1,000 arbitrary units (AU) of K1 killer toxin. Toxin activity, as well as the formation of immunity, was documented after incubation of the plates for 3 days at 20°C (see Fig. S1 in the supplemental material).
