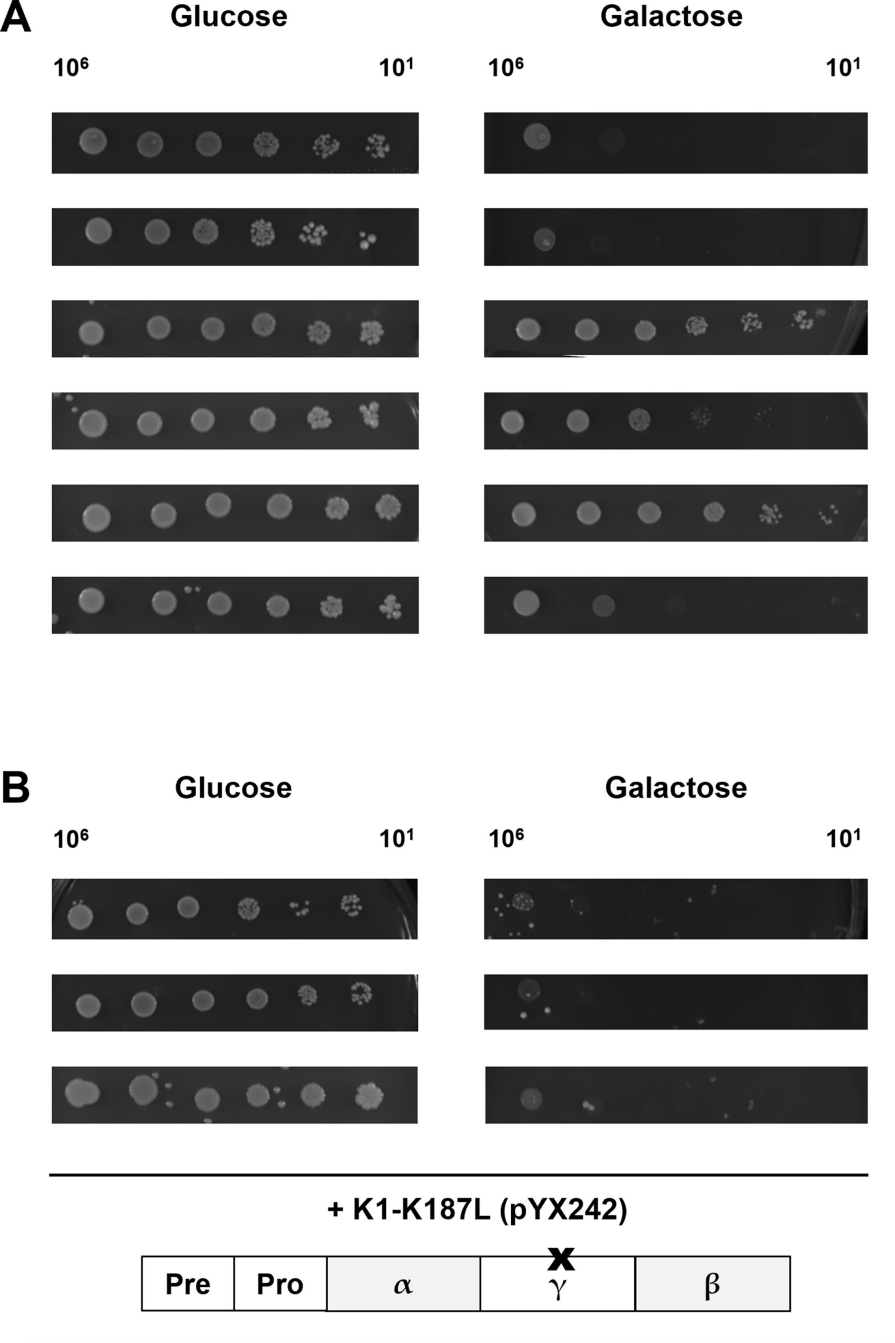# Correction for Gier et al., “Analysis of Yeast Killer Toxin K1 Precursor Processing via Site-Directed Mutagenesis: Implications for Toxicity and Immunity”

**DOI:** 10.1128/msphere.01066-24

**Published:** 2025-01-29

**Authors:** Stefanie Gier, Manfred J. Schmitt, Frank Breinig

## AUTHOR CORRECTION

Volume 5, no. 1, e00979-19, 2020, https://doi.org/10.1128/mSphere.00979-19. Figures 2 and 4 inadvertently included duplicated image parts. To correct these errors, panels have been replaced as detailed below. The revisions do not affect the study’s results, discussion, or conclusions.

Page 4, Fig. 2: Panel A, Glucose row 5 and Galactose rows 2, 3, 5, and 7, and panel B, Glucose rows 1 and 3, should appear as shown in this correction.

**Fig 2:**
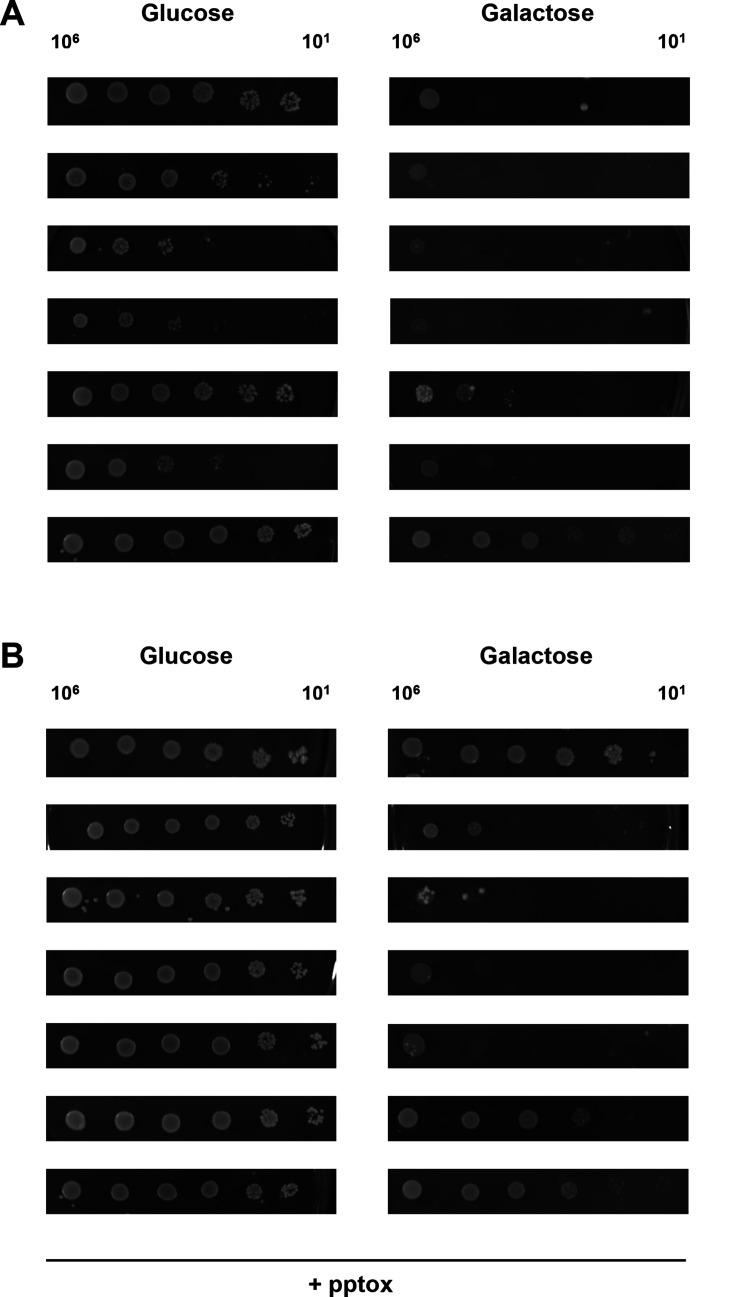


Page 6, Fig. 4: Panel A, Glucose and Galactose row 4, and panel B, Glucose row 1 and Glucose and Galactose row 3, should appear as shown in this correction.

**Fig 4:**