# Supplementary figures and images for: Analysis of Yeast Killer Toxin K1 Precursor Processing via Site-Directed Mutagenesis: Implications for Toxicity and Immunity
Source: mSphere. 2020 Feb 12;5(1):e00979-19. doi: 10.1128/mSphere.00979-19 (PMC7021474; doi:10.1128/mSphere.00979-19)

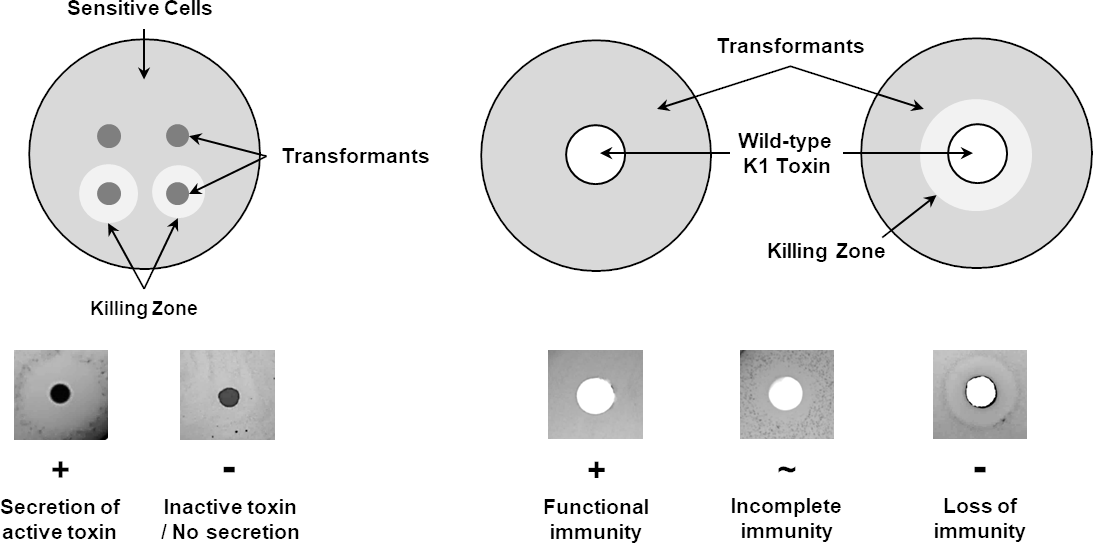

Supplement: FIG S1 [file mSphere.00979-19-sf001.tif]

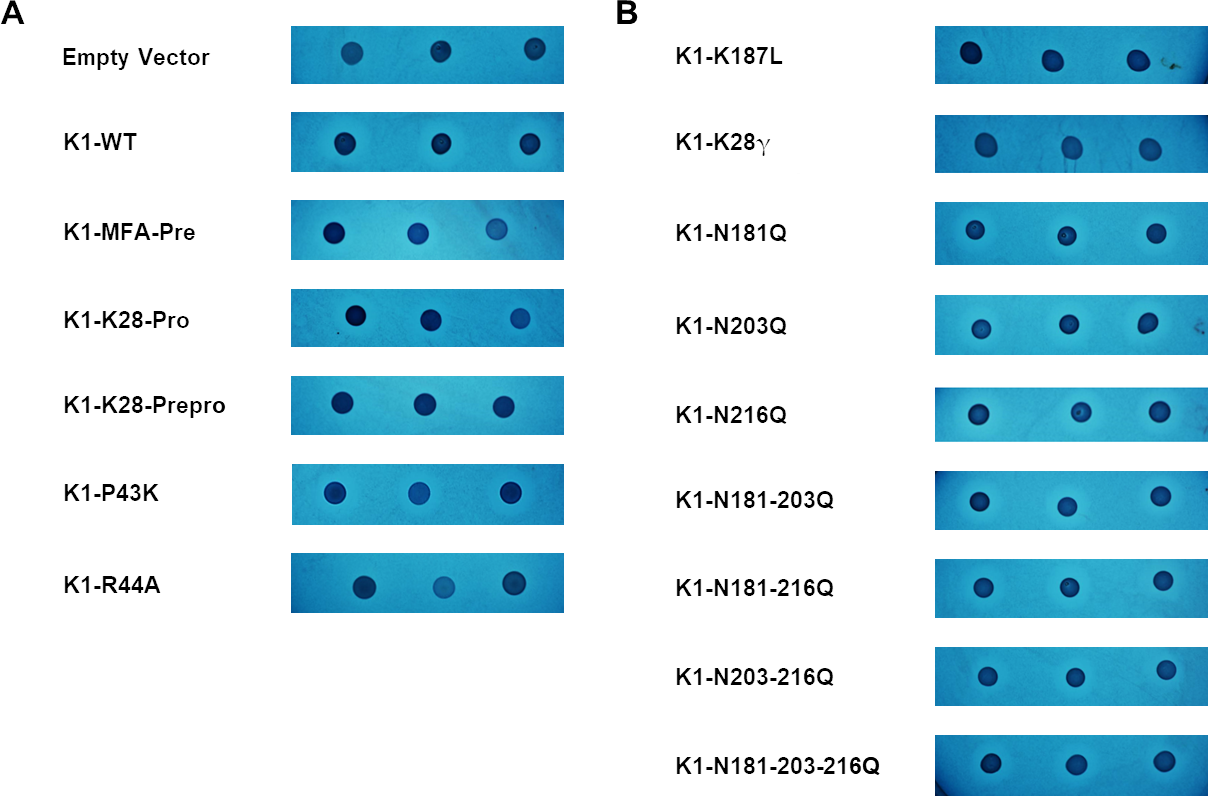

Supplement: FIG S2 [file mSphere.00979-19-sf002.tif]

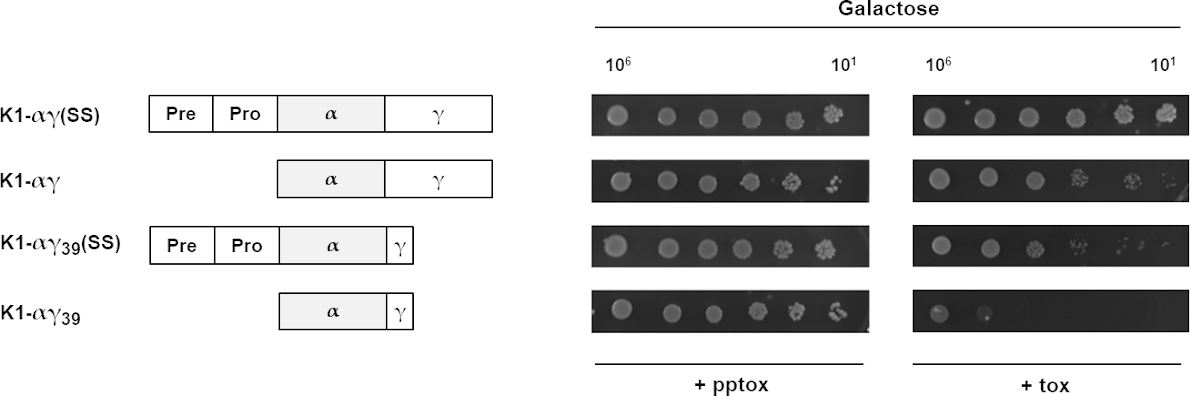

Supplement: FIG S3 [file mSphere.00979-19-sf003.tif]
